# Effectiveness of Prebiotics and Mediterranean and Plant-Based Diet on Gut Microbiota and Glycemic Control in Patients with Prediabetes or Type 2 Diabetes: A Systematic Review and Meta-Analysis

**DOI:** 10.3390/nu16193272

**Published:** 2024-09-27

**Authors:** Nosipho Rosebud Dimba, Nhlakanipho Mzimela, Aubrey Mbulelo Sosibo, Andile Khathi

**Affiliations:** Department of Human Physiology, School of Laboratory Medicine and Medical Sciences, College of Health Sciences, University of KwaZulu-Natal, Westville 3629, South Africa; 218006885@stu.ukzn.ac.za (N.R.D.); 218006756@stu.ukzn.ac.za (N.M.); sosiboa@ukzn.ac.za (A.M.S.)

**Keywords:** prediabetes, type 2 diabetes, gut microbiota, glycemic control, fasting blood glucose, glycated hemoglobin, prebiotics, Mediterranean diet, plant-based diet

## Abstract

Background: A high-calorie diet results in the development of prediabetes (PD) or type 2 diabetes mellitus (T2DM). This diet has been reported to cause changes in microbial composition, concentration levels of glycemic parameters, and immune cells or inflammatory cytokines. This systematic review and meta-analysis aimed to evaluate the effects of prebiotics, as well as Mediterranean and plant-based dietary interventions, on gut microbiota composition and glucose homeostasis in individuals with PD or T2D. Methods: This systematic review and meta-analysis was developed according to the 2020 PRISMA guidelines and checklist. PubMed, EBSCOhost and Google Scholar were the three databases that were used to search for electronically published studies. Data extraction was conducted and examined by the reviewers and all the eligible studies were selected. To test for the quality and biases of the included studies, the Downs and Black checklist was used, followed by the use of Review Manager 5.4. A forest plot was used for meta-analysis and sensitivity analysis. The strength of the evidence was assessed using the Grading of Recommendation Assessment, Development and Evaluation approach. Results: Overall, eight studies met the inclusion criteria: seven focused on patients with T2D, and one focused on patients with PD. The prebiotic dietary intervention did not have a statistically significant effect on glycemic control, including fasting blood glucose (FBG) and glycated hemoglobin (HbA1c). However, one study investigating the Mediterranean diet reported a significant effect on glycemic control. Both prebiotic and Mediterranean dietary interventions were found to beneficially influence gut microbial composition in the intervention groups compared to the placebo groups. No studies assessed the impact of a plant-based diet on microbial composition and glucose parameters. Conclusions: This review indicated that dietary intervention with a prebiotic or Mediterranean diet shows to beneficially improve the gut microbiota composition of *Firmicutes*, *Bacteroidetes* and *Bifidobacteria* in patients with PD or T2D. However, their beneficial effects on FBG and HbA1c were less clear and uncertain due to limited reports, particularly regarding the Mediterranean dietary intervention.

## 1. Introduction

Type 2 diabetes mellitus (T2DM) is a metabolic disorder that causes impaired glucose regulation [1]. This type of diabetes mellitus (DM) is often preceded by prediabetes (PD) [2]. PD is a condition characterized by elevated blood glucose levels that are higher than normal but below the threshold for diabetes diagnosis [3,4]. According to the World Health Organization (WHO), the diagnostic criteria for prediabetes include impaired fasting glucose (IFG) ranging from 6.1 to 6.9 mmol/L and impaired glucose tolerance (IGT) indicated by a 2 h plasma glucose level between 7.8 and 11.0 mmol/L [5]. In contrast, the American Diabetes Association (ADA) defines prediabetes using the same IGT cutoff but sets a lower threshold for IFG at 5.6 to 6.9 mmol/L [6]. Additionally, the ADA includes a glycosylated hemoglobin A1c (HbA1c) level between 5.7% and 6.4% as a diagnostic criterion for prediabetes [4,6]. The prevalence of type 2 diabetes (T2D) is rapidly rising worldwide, becoming a global concern due to its associated comorbidities and increased mortality rate [7]. T2D affects approximately 462 million people worldwide, or 6.28% of the global population [1]. T2D and PD have both been linked to the onset of cardiovascular, renal and gastrointestinal conditions, such as coronary artery disease, diabetic nephropathy and leaky gut syndrome, respectively [8,9]. Moreover, a recent study reported that changes in gut microbiome composition resulting from the chronic consumption of a high-fat, high-carbohydrate diet contribute to the development of PD into T2D [10]. The gut microbiome is composed of diverse microorganisms, including *Firmicutes*, *Bacteroidetes*, *Actinobacteria*, *Proteobacteria* and *Verrucomicrobia* [11]. These microorganisms play a crucial role in overall gut health by preserving the gut lining, reducing inflammation and assisting in nutrient absorption [12,13]. An imbalance in these gut microbial organisms has been associated with various metabolic disorders such as T2D and PD [10,14]. Patients with T2D are reported to have a reduction in butyrate-producing bacteria (*Roseburia* species and *Faecalibacterium prausnitzii*), along with an altered ratio of the major phyla *Firmicutes* to *Bacteroidetes* [15]. This imbalance in bacterial composition is associated with a subclinical pro-inflammatory state, which plays a significant role in the pathogenesis of T2D and contributes to an alteration in glucose homeostasis [15]. However, the use of dietary interventions such as prebiotics and Mediterranean or plant-based diets has shown to be effective in enhancing microbial diversity [16].

Prebiotics are nondigestible food ingredients that have been shown to effectively modulate the gut flora by stimulating the beneficial bacteria in the gut [17,18]. There has been a growing interest in the effect of a Mediterranean diet, as it has been reported to be associated with a lower risk of developing T2D [19]. This diet is high in vegetables, fruit and legumes, but low in red meat [19,20]. A plant-based diet predominantly consists of foods derived from plants such as fruits, vegetables, whole grains, nuts and seeds [21,22]. This diet is associated with a reduced risk of cardiovascular disease and provides additional benefits for gastrointestinal health and glucose homeostasis [21,23,24].

All these types of dietary interventions have been suggested to improve glucose regulation and gut microflora in patients with PD or T2D [17,19,22]. However, the results are inconsistent, especially in human studies. In this systematic review and meta-analysis, we sought to explore the effect of prebiotics, as well as Mediterranean and plant-based dietary interventions, on gut microbial populations and glucose regulation in patients with PD or T2D.

### Study Objectives

To evaluate the effects of dietary interventions (prebiotics, Mediterranean diet and plant-based diet) on fasting blood glucose and glycated hemoglobin (HbA1c) levels in individuals with PD or T2D.

To determine the changes in the gut microbial composition of *Firmicutes*, *Bacteroidetes* and *Bifidobacterium* in individuals with PD or T2D.

## 2. Materials and Methods

The protocol for this systematic review was registered at the International Prospective Register of Systematic Reviews (PROSPERO) (CRD42023478352). This systematic review was developed according to the preferred reporting items for systematic review and meta-analysis (PRISMA) guidelines and the checklist of 2020 [25].

### 2.1. Inclusion and Exclusion Criteria

The inclusion criteria in this study were the following:

(a) Studies that reported individuals with PD or T2D were considered eligible.
(b) Studies that included any of these dietary interventions (prebiotics, Mediterranean diet or plant-based diet)
(c) Studies that reported changes in the gut microbial composition, specifically *Firmicutes*, *Bacteroidetes* and *Bifidobacterium*, in individuals with PD or T2D were considered eligible.
(d) Studies that reported the effectiveness of prebiotics, a Mediterranean diet or a plant-based diet on FBG and HbA1c levels in individuals with PD or T2D.
(e) All categories of study designs were included.
(f) Studies reported between the years 2003 and 2024 were included.

The exclusion criteria were the following:

(a) We excluded all animal studies, editorials, letters and case studies.
(b) Studies with insufficient details on the methodology and findings were excluded.
(c) Studies reported before the year 2003 were excluded.

### 2.2. Data Sources and Search Strategy

The study search was conducted using three electronic databases: PubMed, EBSCOhost and Google Scholar. Medical search headings (MeSHs) were used in the formulation of a systematic search strategy, using keywords such as “prediabetes OR type 2 diabetes” AND “prebiotics OR prebiotic diet”, OR “Mediterranean diet OR Mediterranean-style diet”, OR “plant-based diet” AND “glycaemic control OR glucose homeostasis OR HbA1c OR glycated haemoglobin OR fasting blood glucose” AND “gut microbiome OR gut microflora OR gut bacteria” (see Table S1).

### 2.3. Study Selection

Zotero software version 6.0.36 was utilized to manage the studies gathered from the three databases following the search strategy, as well as to exclude duplicate studies. After the removal of duplicates, the titles and abstracts of the remaining articles were screened according to the eligibility criteria by three independent authors (NRD, NM and AMS). The full texts were also screened by these authors and the studies that met the inclusion criteria were selected. Conflicts were settled by a fourth author (AK).

### 2.4. Data Collection Process

Data extraction was executed by three authors independently (NRD, NM and AMS), and the fourth author (AK) was approached in case of any disagreements between the three authors. All important information, such as authors, country, year of publication, study design, title, number of participants, characteristics of individuals with PD or T2D (e.g., age), study duration, type of intervention, type of control, glycemic control parameters, gut microbiota abundance and diversity, were recorded in a Microsoft Excel 365 table created by three authors (NRD, NM and AMS). The fourth author (AK) read and approved the content of this Microsoft Excel table. The studies included in the analysis examined primary outcomes related to the impact of dietary interventions on gut microbial composition (notably *Firmicutes*, *Bacteroidetes* and *Bifidobacteria*) and glycemic levels, specifically FBG and HbA1c. 

### 2.5. Study Risk of Bias Assessment

The Downs and Black checklist was used to measure the bias in each eligible study (see Supplementary Materials). The checklist was based on 4 domains: report of bias (10 items), external validity (3 items), internal validity (6 items) and selection bias (7 items). The scores were rated as follows: excellent (25), good (20–24), moderate (14–19), poor (11–13) and very poor (<10). Three independent authors (NRD NM and AMS) were assigned the responsibility of assessing each study independently based on these four domains. Any disagreements were resolved by consulting the fourth author (AK). The overall score rate in the meta-analysis was excellent. 

### 2.6. Data Synthesis and Analysis

The meta-analysis was performed using Review Manager version 5.4 software (RevMan). Using the forest plot in this software, eligible data from all the included studies were analyzed using the mean and standard deviation of FBG and HbA1c in both the intervention and control/placebo groups. To quantify the size of the connection, the forest plot provided a mean difference and confidence interval, with solid lines denoting the 95% confidence interval. Each eligible study was represented as a horizontal line on the *y*-axis to list the primary author and year of study. The forest plot also included the weight of the study results, which was automatically detected or calculated by the RevMan 5.4 software. A random-effects model was used on the assumption that the effects being estimated in the different studies were not identical and that the data followed a normal distribution. Heterogeneity among the studies was also evaluated using the RevMan 5.4 forest plot, *I*^2^, Chi-square and a statistical test. The *I*^2^ statistic provides an estimate of the extent of inconsistency attributable to inter-study heterogeneity. Studies with *I*^2^ values of 25, 50 and 75% indicate low, moderate and high levels of heterogeneity, respectively. For the results that showed substantial and high heterogeneity, we explored potential possible causes through a subgroup analysis and sensitivity analysis when required.

### 2.7. Certainty Assessment of Strength of Evidence

The four authors (NRD, NM, AMS and AK) assessed the quality of evidence across the selected studies using the Grading of Recommendation Assessment, Development and Evaluation (GRADE) approach [26,27]. Five GRADE considerations (risk of bias, consistency of effect, imprecision, indirectness and publication bias) were utilized to explore the certainty of evidence (high, moderate, low and very low).

## 3. Results

### 3.1. Study Selection

**Figure 1:**
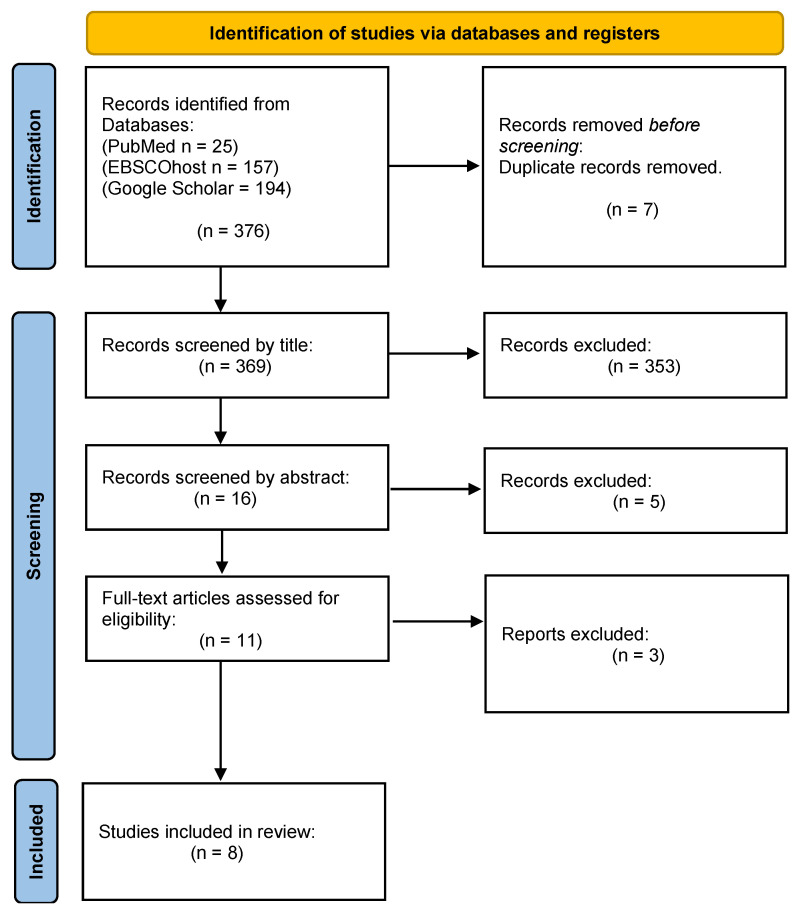
Flowchart of the study selection process.

Our primary search identified 376 studies through the three electronic search databases. However, for this systematic and meta-analysis, only eight studies were considered eligible, as Figure 1 illustrates below [28,29,30,31,32,33,34,35]. A total of 7 duplicate studies were removed and 353 did not satisfy the inclusion criteria according to the title. Additionally, 16 articles were excluded after reviewing their abstracts. The other three studies did not have full text.

### 3.2. Study Characteristics

All the included studies were published in peer-reviewed journals between 2003 and 2024, and their characteristics are shown in Table 1. Briefly, this study had a total of 488 participants, with mean ages of 54.3 ± 9.7; 55 ± 11; 58.1 ± 1.7; 59.2 ± 7.2; 48.69 ± 9.74; 54.4 ± 8.3; 66 ± 9 and 63.1 years. Seven studies investigated individuals with T2D [28,29,31,32,33,34,35] and one study investigated individuals with PD [30].

### 3.3. Risk of Bias Assessment

The three authors NRD, NM and AMS assessed the risk of bias and quality of the included studies using a modified Downs and Black checklist [36]. Six included studies rated excellent (25 points) while the other two rated good (20–24 points) (see Table S2). The overall quality of the included studies in this meta-analysis was excellent.

### 3.4. Data Synthesis and Analysis

#### 3.4.1. Effect of Prebiotic Interventions on Glycemic Control Parameters

Fasting blood glucose (mmol/L) levels

**Figure 2:**
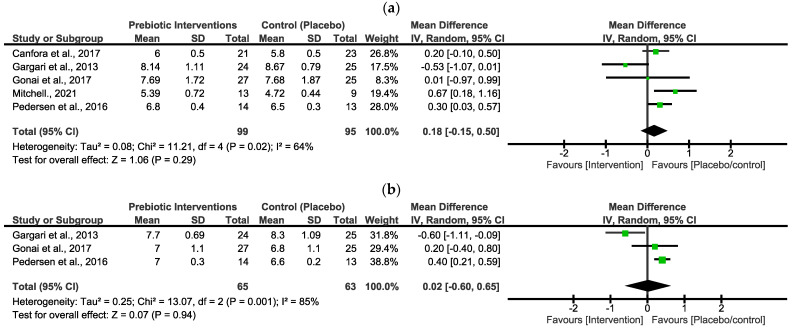
Forest plots assessing the effects of prebiotic interventions in FBG (**a**) [29,30,33,34,35] and HbA1c (**b**) [29,34,35].

In Figure 2a, the pooled effect estimates of FBG indicated a non-statistical difference between the intervention group and the placebo/control group. However, the overall estimates favoured the placebo/control group [MD = 0.18 (−0.15, 0.50), *p* = 0.29]. There was a moderate value of statistical heterogeneity in these studies (x^2^ = 11.21, *I*^2^ = 64%, *p* = 0.02).

Glycated hemoglobin (%) levels

In Figure 2b, the pooled effect estimates of HbA1c indicated a non-statistical difference between the intervention group and the placebo/control group. The overall estimates did not favour either group [MD = 0.02 (−0.60, 0.65), *p* = 0.94]. There was a high statistical heterogeneity in these studies (x^2^ = 13.07, *I*^2^ = 85%, *p* = 0.001).

Prebiotic intervention subgrouping and sensitivity analysis

**Figure 3:**
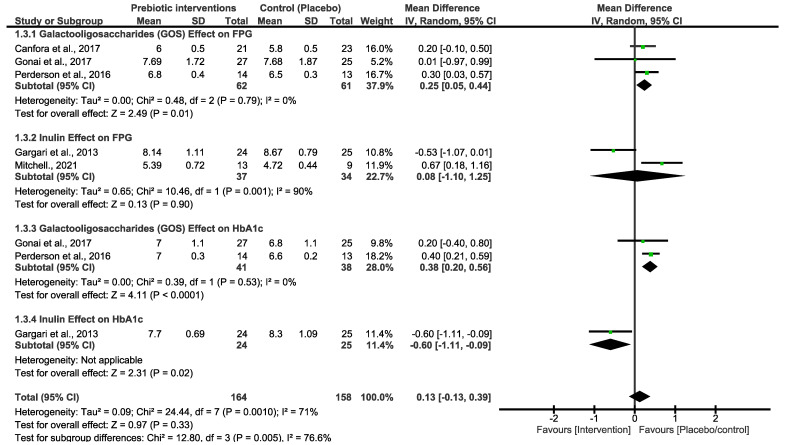
Forest plot subgrouping assessing different types of prebiotic interventions in FBG [29,30,33,34,35] and HbA1c [29,34,35].

**Figure 4:**
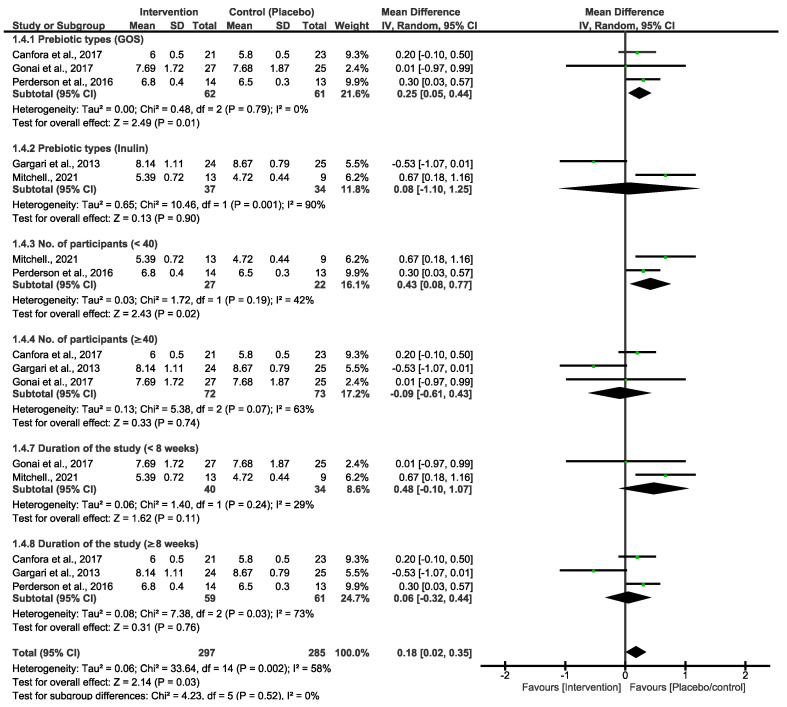
Subgroup analyses for the primary prebiotic interventions of FBG [29,30,33,34,35].

Both primary outcomes resulted in moderate and high heterogeneity; we therefore performed a subgroup analysis using different types of prebiotic interventions (GOS and inulin) to explore variations in FBG and HbA1c. As shown in Figure 3, the pooled effect estimates of GOS and inulin subgrouping indicated a non-statistical difference between the prebiotic intervention group and the placebo/control group. However, the overall estimates favoured the placebo/control group [MD = 0.13 (−0.13, 0.39), *p* = 0.33]. There was high heterogeneity in these studies (x^2^ = 24.44, *I*^2^ = 71%, *p* = 0.0010). To further explore heterogeneity, we subgrouped the primary outcomes of FBG by prebiotic types, number of participants and duration of the studies, as shown in Figure 4. These analyses showed a slight change in the effect estimate and heterogeneity [MD = 0.18 (0.02, 0.35), *I*^2^ = 58%, *p* = 0.03]. In a sensitivity analysis, the overall effect estimate remained insignificant when the subgrouping analysis was conducted for the second time with a fixed effect model. Moreover, the pooled effect continued to favour the placebo/control group when any of the studies were omitted in this subgrouping. Excluding the studies that utilized inulin as the intervention diet led to decreased heterogeneity [33,34]. 

#### 3.4.2. Effects of Prebiotics and Mediterranean Dietary Intervention on Gut Microbiota Composition

According to the results presented in Table 2, seven of the eligible studies included in this systematic review and meta-analysis reported on the effects of various prebiotic dietary interventions, such as galactooligosaccharides (GOS) and inulin-type fructans, on bacterial abundance and diversity. In a study conducted by [29,35], the GOS prebiotic type significantly increased the abundance of *Bifidobacteriaceae* in the intervention group in comparison to the placebo/control group in individuals with T2D [29,35]. Canfora et al. 2017 [30], discovered that GOS increased the abundance of *Bifidobacterium* in the intervention group relative to the placebo group among men and women with PD [30]. However, GOS did not affect the microbial diversity in this study [30]. The prebiotic (inulin-type fructans) significantly increased the abundance of *Bifidobacteriaceae* and *Bacteroidetes* in the intervention group of patients with T2D, but the microbial diversity was not affected by the inulin-type fructans [33,34]. The prebiotic fibre-enriched nutritional formula was shown to be effective in significantly increasing the *Bifidobacteriaceae* and *Firmicutes* in the intervention group versus the non-blinded dietary group. The eighth eligible study reported the effect of Mediterranean dietary intervention [31]. The overall outcomes by Ismael et al. 2021 [31], indicated that the use of this diet increased bacterial richness after 4 and 12 weeks, and the *Prevotella*-*to*-*Bacteroidetes* ratio was also reported to have increased (Table 2).

### 3.5. Certainty of Evidence

For the prebiotic interventions, only five studies were included for the glycemic marker, namely FBG and the GRADE certainty evidence of this marker resulted in a low score of (2/4) due to limitations in the study design, execution and inconsistency (substantial average statistical heterogeneity) [29,30,33,34,35]. For HbA1c, three studies (prebiotic interventions) were included, and GRADE certainty evidence for HbA1c resulted in a very low score (1/4) due to limitations in study design, execution and inconsistency (strong statistical heterogeneity) [29,34,35]. The quality of the included evidence, as well as the meta-analysis summary of the findings, is reported in Table 3. For the Mediterranean intervention, heterogeneity of both FBG and HbA1c could not be calculated because there was only one study that reported this type of dietary intervention in individuals with T2D [31].

## 4. Discussion

According to a previous study, the overconsumption of high-calorie diets dysregulates glucose homeostasis and the balance of microbial diversity or abundance in the gut [10]. The alteration in these two factors has been shown to increase the risk of occurrence and development of PD or T2D and its associated complications [10,14]. Therefore, this study aimed to systematically review the effectiveness of different types of dietary interventions, which have been shown to restore gut microbiome composition as well as regulate glycemic control in patients with PD or T2D [19,23]. Three dietary interventions were reviewed, including prebiotics, Mediterranean diets and plant-based diets. Prebiotics are nondigestible food ingredients that beneficially affect the host by selectively stimulating the growth activity of one or a limited number of beneficial bacteria in the colon, thus improving host health [37]. Prebiotics include galactooligosaccharides (GOS), large polysaccharides (inulin/cellulose), maltooligosaccharides (MOS) and fructooligosaccharides (FOS) [17,38]. The Mediterranean diet is a plant-based dietary pattern that sees the majority of its protein coming from legumes, nuts or grains supplemented with fresh seafood and a low amount of red meat [39,40]. This type of diet also typically includes fruit, vegetables and healthy fats from avocados and olive oil that have no negative effect on one’s overall health [20]. A plant-based diet is more of a dietary lifestyle that emphasizes whole, minimally processed foods [41]. It focuses on different types of plants and excludes refined foods like sugars, processed food and animal products high in calories [22].

The markers that were eligible for this study were FBG, HbA1c and gut microbial composition, such as (*Firmicutes*, *Bacteroidetes* and *Bifidobacteria*). These bacteria were of interest as they are the most dominant gut bacteria in humans and they play a significant role in the digestion of food, stimulation of cell growth and strengthening of the immune system [11,12]. This systematic and meta-analysis identified eight studies that met the inclusion criteria [28,29,30,31,32,33,34,35]. However, these studies reported the effect of a Mediterranean diet and different types of prebiotic dietary interventions [28,29,30,31,32,33,34,35]. No studies were found that reported the effect of a plant-based dietary intervention on glycemic control and gut microbiota composition.

The first objective of this study was achieved by conducting a meta-analysis of the markers, namely, FBG and HbA1c. The forest plot in Figure 1 includes studies that investigated patients with T2D [29,30,34,35] and in a study conducted by [33] individuals with prediabetes were investigated [33]. In the synthesized results, FBG did not significantly decrease with the use of the prebiotic interventions (Figure 1). The intervention group was also not favoured in comparison to the placebo/control group. These outcomes suggest that prebiotic interventions are not effective in improving glycemic control in patients with PD or T2D. In addition, the certainty evidence of FBG outcomes was low (GRADE score = 2/4), and there was a substantial average heterogeneity (*I*^2^ = 64%) for all five included studies [29,30,33,34,35]. The outcomes in Figure 2 for HbA1c revealed that both the prebiotic interventions and the placebo/control group were not favoured. The synthesized results in the current study further imply that prebiotic dietary interventions did not significantly improve glycemic regulation or control in patients with T2D. The three included studies also showed a very low certainty evidence (GRADE score = 1/4), and plus there was a strong heterogeneity (*I*^2^ = 85%) for HbA1c. The implications of both of these findings suggested that a subgrouping was to be attempted to explore the possible cause of this variation among these studies. 

The studies in Figure 3 are singly subgrouped to different types of prebiotic interventions to explore heterogeneity. Three of the studies that used GOS demonstrated low heterogeneity. Canfora et al. 2017 and Pedersen et al. 2016 [30,35] both used a 12-week intervention period, whereas [29] employed a shorter 4-week experimental period. Nonetheless, none of these studies showed a significant effect of GOS at the end of the experimental period on either of the glycemic control parameters. For inulin intervention, two studies demonstrated a very strong heterogeneity (90%), and the synthesized results were suggested to be due to the varying length between the two studies. Ref. [33] took 6 weeks, and their inulin intervention was not effective. Interestingly, in a study conducted by [34], 8 weeks of the experimental period was significantly effective in reducing the FBG and HbA1c in patients with T2D. Overall, inulin was shown to be more effective than GOS, and these two studies are the cause of the high heterogeneity that is observed in both Figure 1 and Figure 2. In addition, the varying lengths of interventions across the studies made it difficult to determine a more certain and long-term effect of GOS and inulin on FBG and HbA1c. The actual impact of these different types of prebiotic dietary interventions on FBG and HbA1c may differ from what our investigation revealed. The results displayed in Figure 4, which include additional subgroup analyses, indicated that studies with small sample sizes (<40) and shorter durations (<8 weeks) exhibited moderate heterogeneity compared to those with larger sample sizes (≥40) and longer durations (≥8 weeks). The results of this subgroup analysis showed no significant changes, indicating high sensitivity. Consequently, there is limited confidence in the effect estimates for FBG, suggesting that the true effect may differ from the estimated effect. This is further supported by a low GRADE score for the certainty of evidence regarding FBG outcomes. These results suggest that the true effect of these dietary interventions in FBG may be different from what this review depicted. Moreover, further studies with a reduced risk of bias are required to produce higher certainty of evidence to reach more reliable results than this study.

The meta-analysis and forest plots for the Mediterranean dietary intervention could not be synthesized as there was only one study that was found eligible for our inclusion criteria. The findings reported by [31] indicated a significant reduction in FBG in patients with T2D following 4 to 12 weeks of a Mediterranean dietary intervention. After 12 weeks with the use of a Mediterranean diet, a significant decrease in HbA1c in patients with T2D in comparison to the baseline was reported. Overall, the results of [31] study suggested that this diet may exert a significant effect on glycemic control or regulation.

The human gut microbiome comprises over three trillion bacteria, which play a significant role in our health [42,43]. Disturbances of the healthy symbiotic relationship between the human gut and gut microbiota result in dysbiosis, which is associated with PD or T2D [44]. Therefore, improving the abundance and diversity of beneficial bacteria using dietary intervention has been recommended recently as a control or prevention method for PD or T2D [45]. These reports brought us to the second aim of this study, which was achieved by assessing the prebiotic and Mediterranean diet effect on different types of bacterial compositions. The results revealed that a GOS prebiotic type significantly increased the abundance of *Bifidobacterium* in patients with PD and T2D compared to those receiving a placebo or control treatment [29,35]. In a study conducted by [30], the GOS prebiotic type also showed no significant increase in microbial diversity, and there was only one type of microbial species that was included in the findings of their study [30]. Inulin-type fructans significantly increased not only the *Bifidobacterium* species but also the *Bacteroidetes* in the prebiotic intervention group in comparison to the placebo/control group [32,33]. However, the inulin-type fructans did not affect the other microbial species that form part of the inclusion criteria (*Firmicutes*). Another type of prebiotics is fibre-enriched nutritional formula, which showed an increased significance of *Bifidobacterium* and *Firmicutes* in the intervention group compared to other dietary groups/placebo [28]. The findings of these studies confirmed that the use of prebiotics enhances microbial growth in the gastrointestinal tract, while the Mediterranean dietary intervention increased bacterial richness in individuals with T2D after 4–12 weeks of the intervention period [31,37].

### 4.1. Study Limitations

This systematic review and meta-analysis comprises seven studies that investigated prebiotic interventions. However, in one of these studies, the mean and standard deviation data for FBG and HbA1c could not be retrieved to conduct a forest plot in RevMan 5.4. The author of this study was contacted but the email account did not exist. The application data of these results would have been utilized to gain a more thorough understanding of these prebiotic interventions.

### 4.2. Recommendations

Further research is required to conduct comparative analyses of various interventions and their dosages, aimed at delineating their relative efficacy in modulating glucose metabolism among individuals with T2D or PD. Moreover, the investigation of prolonged interventions with these dietary regimens is of interest, given that a study meeting the inclusion criterion demonstrated beneficial effects on glycemic parameters over 8 weeks of inulin intervention.

## 5. Conclusions

The different types of prebiotics and the Mediterranean diet used in the studies were shown to positively impact the gut microbiome in patients with PD or T2D. However, the prebiotic dietary intervention did not significantly reduce FBG or HbA1c levels in these patients. A low and very low certainty of evidence for FBG and HbA1c was also reported. Additionally, while the Mediterranean diet has been shown to be effective in reducing FBG and HbA1c, the overall results are uncertain due to the fact that only one study has reported on this dietary intervention. These outcomes also highlighted the limited evidence regarding the potential mechanisms by which prebiotics and the Mediterranean diet may improve glucose regulation. Therefore, additional studies are needed to further evaluate these dietary interventions and the impact of a plant-based diet on glucose parameters and other microbial species in patients with PD and T2D.

## Figures and Tables

**Table 1:** Summary characteristics of the included studies.

Study	Study Design	Location of the Study	Participants	Age (Sex)	Study Duration	Type of Intervention	Type of Control	Experimental Outcomes for Glucose Metabolism Parameters	Experimental Outcomes for Gut Microbiota
[28]	Randomized Controlled Trial	USA	192	54.3 ± 9.7 years (F and M)	12 weeks	prebiotic fibre-enriched nutritional formula (active)	A placebo fibre-absent nutritional formula (placebo), or non-blinded dietary advice alone (diet)	At week 12, HbA1c was reduced significantly in active vs. placebo. In the three study arms (active, placebo and diet), FBG at week 12 did not change significantly from baseline and there was no difference in the change between the arms.	*Roseburia bromii (Firmicutes) and Bifidobacterium* spp. increased in the active arm versus the diet arm. *Roseburia faecis (Firmicutes)* and *Anaerostipes hadrus (Firmicutes)* increased significantly in active vs. placebo (*p* = 0.0037 and *p* = 0.043, respectively).
[31]	Non-Randomized Controlled Trial	Portugal	9	66 ± 9 years (F and M)	4 weeks and 12 weeks	Mediterranean diet	No comparison group (baseline and endpoint was used)	FBG at baseline decreased after 12 weeks of the experimental period. Twelve weeks after the intervention, HbA1c significantly decreased by 0.67% (*p* < 0.05).	*Prevotella*-to-*Bacteroidetes* ratio tended to increase right after 4 weeks of the intervention period. The diversity and richness of *Firmicutes*-to-*Bacteroidetes* ratio was shown to be decreased after the intervention, as these patients had a higher ratio of these phyla.
[34]	Randomized Controlled Trial	Iran	49	48.69 ± 9.74 years and 47.77 ± 10.14 years	8 weeks	Inulin-type fructans supplementation	Maltodextrin	After 8 weeks, there was a significant decrease in the FBG (8.47%) and HbA1c (10.43%) in the inulin group when compared to that of the maltodextrin group (*p* < 0.05).	-
[29]	Randomized Controlled Trial	Japan	116	55± 11 years and 54 ± 12 years	4 weeks	GOS (prebiotics)	Placebo group	FBG and HbA1c did not improve after consumption of GOS and there were no significant changes that were observed after the experimental period.	*Bifidobacteriaceae*, *Firmicutes* and *Bacteroidetes* abundances (%) were significantly increased in GOS compared to in the placebo group (*p* < 0.05).
[35]	Randomized Controlled Trial	UK	29	56.7 ± 1.6 years and 58.1 ± 1.7 years	12 weeks	Prebiotic (GOS mixture)	Placebo (maltodextrin) supplement	Prebiotic treatment had no significant effect on FBG and HbA1c concentrations. However, the changes in glucose effectiveness at zero insulin in the placebo group was significantly different from the prebiotic group.	*Bifidobacterium* levels increased in both groups; however, the change within the prebiotic group was greater and close to significance (*p* = 0·0582). Prebiotic treatment had no significant effect on total bacteria, *Firmicutes (Lactobacillus, Roseburia, Enterobacteriaceae, Clostridium)* groups
[32]	Randomized Controlled Trial	Norway	25	63.1 (41–73) years	6 weeks	Inulin-type fructans (a mixture of oligofructose and inulin)	(placebo) Maltodextrin	-	Six weeks of supplementation with inulin-type fructans had a significant increase in the bacterial composition of *Bifidobacterium adolescentis*, followed by *Bacteroidetes*.
[33]	Randomized Controlled Trial	USA	24	54.4 ± 8.3 years	6 weeks	Prebiotic Inulin	(placebo) Maltodextrin	FBG did not change (*p* > 0.05) with the intervention in the placebo.	*Bifidobacteria* increased with inulin supplementation but not in the placebo group (*p* = 0.04)
[30]	Randomized Controlled Trial	The Netherlands	44	59.2 ± 7.2 years and 58.4 ± 7.3 years	12 weeks	Prebiotic (GOS)	isocaloric placebo (maltodextrin)	No changes in FBG were observed between treatments.	Twelve-week GOS supplementation consistently increased fecal *Bifidobacterium* compared with placebo

Abbreviations: GOS (galactooligosaccharides); FBG (fasting blood glucose); USA (United States of America); HbA1c (glycated hemoglobin); UK (United Kingdom); F (female); M (male).

**Table 2:** Result summary of the eligible studies that investigated the effects of different types of prebiotic interventions on gut microbiota diversity and abundance.

Study	Type of Intervention	Diversity	Gut Microbiota Composition	Change in Relative or Absolute Abundance
[32]	Inulin-type fructans VS. maltodextrin as the control group	Microbial diversity was not affected by the prebiotic fibre after the 6-week intervention	1. *Bifidobacterium adolescentis* 2. *Bacteroidetes* 3. *Firmicutes*	*Bifidobacterium adolescentis* had the largest effect size in the intervention group. However, the *Bacteroidetes or Firmicutes* had a smaller effect size when compared to the *bifidobacterium*.
[30]	Prebiotic (GOS) VS. isocaloric placebo (maltodextrin)	The overall microbial richness (P ¼ 0.307) and diversity (P ¼ 0.626) were not different between groups	1. *Bifidobacterium*	Supplementation of the prebiotic galactooligosaccharides increased the *Bifidobacterium* species abundance by 5.0- ± 0.3-fold (P ¼ 0.009) q ¼ 0.144) compared with the placebo.
[28]	Prebiotic fibre-enriched nutritional formula (active) VS. a placebo fibre-absent nutritional formula (placebo), or non-blinded dietary advice alone (diet)	No significant changes in alpha diversity were observed from baseline to the end of the intervention for any groups. A small but significant portion of the variance in beta diversity was observed in the active arm relative to the control arms (R^2^ = 0.012, *p* < 0.001, adonis test)	1. *Bifidobacterium* 2. *Roseburia faecis* and *Anaerostipes hadrus (Firmicutes)*	1. The relative abundance of *Bifidobacterium adolescentis* increased in the active arm versus the diet arm, albeit not significantly (*p* = 0.19). 2. The relative abundance of *Roseburia faecis* and *Anaerostipes hadrus* increased significantly from baseline to end of intervention in the active arm relative to the diet arm (*p* = 0.0037 and *p* = 0.043, respectively).
[29]	Prebiotics (GOS) VS. placebo group	-	1. *Bifidobacteriaceae* 2. *Firmicutes* 3. *Bacteroidetes*	*Bifidobacteriaceae* abundance increased by the intake of GOS intervention compared with the baseline (35.2 ± 23.4 vs. 12.8 ± 14.5%, *p* < 0.05). *Firmicutes* and *Bacteroidetes* showed no significant difference between the GOS group and the placebo group.
[31]	Mediterranean diet	No differences were observed in bacterial diversity by the end of the intervention (1.80 ± 0.75 to 2.05 ± 0.47, *p* = 0.449; Cohen d = 0.40)	1. *Firmicutes* 2. *Bacteroidetes* 3. *Bifidobacterium*	Bacterial richness increased from baseline to 4 and 12 weeks after the intervention.
[33]	Inulin-type fructans VS. maltodextrin as the control group	*Bifidobacteria* increased with inulin supplementation but not in the placebo group (*p* = 0.04)	1. *Bifidobacterium*	*Bifidobacteria* 16s increased (*p* = 0.04; inulin = ∆3.19, placebo = ∆−8.9 × 10^8^) with inulin supplementation.
[35]	Prebiotic (GOS) mixture VS. placebo (maltodextrin) supplement	Prebiotic fibre treatment did not induce significant changes in diversity when compared with the placebo group	1. *Bifidobacterium* 2. *Firmicutes (Lactobacillus, Roseburia, Enterobacteriaceae, Clostridium)*	Prebiotic fibre treatment did not induce significant changes in the relative abundance of species and richness (the number of species per sample) indices when compared with the placebo group.

Abbreviations: GOS (galactooligosaccharides).

**Table 3:** Certainty of evidence (GRADE) of the summarized meta-analysis findings.

Certainty Assessment							№ of Patients		Effect		Certainty	Importance
№ of Studies	Study Design	Risk of Bias	Inconsistency	Indirectness	Imprecision	Other Considerations	Prebiotic Interventions	Placebo/Control	Relative (95% CI)	Absolute (95% CI)		
Outcome 1: Fasting blood glucose												
5	randomized trials	Serious ^a^	Serious ^b^	not serious	not serious ^c^	none	99	95	-	MD 0.18 higher (0.15 lower to 0.5 higher)	⨁⨁◯◯ Low	IMPORTANT
Outcome 2: Hemoglobin (HbA1c)												
3	randomized trials	Serious ^a^	very serious ^d^	not serious	not serious ^c^	none	65	63	-	MD 0.02 higher (0.6 lower to 0.65 higher)	⨁◯◯◯ Very low	IMPORTANT

Note: CI, confidence interval; MD, mean difference. ^a.^ The eligible studies did mention their serious limitations at the end of their papers. ^b.^ There was an average value of statistical heterogeneity in these studies that measured FBG. ^c.^ CI crosses the clinical decision threshold between the intervention group and the placebo/control group. ^d.^ There was substantial, strong statistical heterogeneity in these studies that measured HbA1c.

## Supplementary Materials

The following supporting information can be downloaded at: https://www.mdpi.com/article/10.3390/nu16193272/s1, Table S1: PUBMED search strategy; Table S2: Downs and Black checklist.
