# Supplementary material for: Effectiveness of Prebiotics and Mediterranean and Plant-Based Diet on Gut Microbiota and Glycemic Control in Patients with Prediabetes or Type 2 Diabetes: A Systematic Review and Meta-Analysis
Source: Nutrients. 2024 Sep 27;16(19):3272. doi: 10.3390/nu16193272 (PMC11478454; doi:10.3390/nu16193272)
Supplement: Supplementary file 1 [file nutrients-16-03272-s001.zip › nutrients-3192590-supplementary.pdf]

Supplementary Table S1. PUBMED Search Strategy

SEARCH DETAILS	RESULTS
**((("diet, Mediterranean"[MeSH Terms] OR "Prebiotics"[MeSH Terms] OR "diet, plant-based"[MeSH Terms]) AND "diabetes mellitus, type 2"[MeSH Terms]) OR "Prediabetic State"[MeSH Terms]) AND "Gastrointestinal Microbiome"[MeSH Terms]**	147
**(((("diet, Mediterranean"[MeSH Terms] OR "Prebiotics"[MeSH Terms] OR "diet, plant-based"[MeSH Terms]) AND "diabetes mellitus, type 2"[MeSH Terms]) OR "Prediabetic State"[MeSH Terms]) AND "Gastrointestinal Microbiome"[MeSH Terms]) AND (humans[Filter])**	118
**(((("diet, mediterranean"[MeSH Terms] OR "Prebiotics"[MeSH Terms] OR "diet, plant based"[MeSH Terms]) AND "diabetes mellitus, type 2"[MeSH Terms]) OR "Prediabetic State"[MeSH Terms]) AND "Gastrointestinal Microbiome"[MeSH Terms]) AND ((clinicaltrial[Filter] OR randomizedcontrolledtrial[Filter]) AND (humans[Filter]))**	25

Supplementary Table S2. Downs and Black Checklist

	Author Name and Reference	**Frias *et al.*, 2023 [28]**	**Gonai *et al.*, 2017 [29]**	**Pedersen *et al.*, 2016 [35]**	**Canfora *et al.*, 2017 [30]**	**Birkel and *et al.*, 2020 [32]**	**Mitchell *et al.*, 2021 [33]**	**Gargari *et al.*, 2013 [34]**	**Ismael *et al.*, 2021 [31]**
	**Reporting**								
1	*Is the hypothesis/aim/objective of the study clearly described?*	1	1	1	1	1	1	1	1
2	*Are the main outcomes to be measured clearly described in theIntroduction or Methods section?*<br>If the main outcomes are first mentioned in the Results section, thequestion should be answered no.	1	1	1	1	1	1	1	1
3	*Are the characteristics of the patients included in the study clearlydescribed?*<br>In cohort studies and trials, inclusion and/or exclusion criteria should begiven. In case-control studies, a case definition and the source for controls should be given.	1	1	1	1	1	1	1	1
4	*Are the interventions of interest clearly described?*<br>Treatments and placebo (where relevant) that are to be compared shouldbe clearly described.	1	1	1	1	1	1	1	1
5	*Are the distributions of principal confounders in each group of subjects tobe compared clearly described?*<br>A list of principal confounders is provided.0=No. 1=partially. 2= yes.	0	2	2	2	2	2	2	0
6	*Are the main findings of the study clearly described?*<br>Simple outcome data (including denominators and numerators) should bereported for all major findings so that the reader can check the major analyses and conclusions. (This question does not cover statistical tests which are considered below).	1	1	1	1	1	1	1	1

7	*Does the study provide estimates of the random variability in the data forthe main outcomes?* In non normally distributed data the inter-quartile range of results should be reported. In normally distributed data the standard error, standard deviation or confidence intervals should be reported. If the distribution ofthe data is not described, it must be assumed that the estimates used were appropriate and the question should be answered yes.	1	1	1	1	1	1	1	1
8	*Have all important adverse events that may be a consequence of theintervention been reported?* This should be answered yes if the study demonstrates that there was acomprehensive attempt to measure adverse events. (A list of possible adverse events is provided).	1	1	1	1	1	1	1	1
9	*Have the characteristics of patients lost to follow-up been described?* This should be answered yes where there were no losses to follow-up or where losses to follow-up were so small that findings would be unaffected by their inclusion. This should be answered no where a studydoes not report the number of patients lost to follow-up.	1	1	1	1	1	1	1	1
10	*Have actual probability values been reported(e.g. 0.035 rather than <0.05) for the main outcomes except where the probability value is lessthan 0.001?*	1	1	1	1	1	1	1	1

	External validity								
11	*Were the subjects asked to participate in the study representative of theentire population from which they were recruited?*<br>The study must identify the source population for patients and describe how the patients were selected. Patients would be representative if they comprised the entire source population, an unselected sample of consecutive patients, or a random sample. Random sampling is only feasible where a list of all members of the relevant population exists. Where a study does not report the proportion of the source population from which the patients are derived, the question should be answered asunable to determine.	1	1	1	1	1	1	1	1
12	*Were those subjects who were prepared to participate representative ofthe entire population from which they were recruited?*<br>The proportion of those asked who agreed should be stated. Validation that the sample was representative would include demonstrating that thedistribution of the main confounding factors was the same in the study sample and the source population.	1	1	1	1	1	1	1	1
13	*Were the staff, places, and facilities where the patients were treated, representative of the treatment the majority of patients receive?*<br>For the question to be answered yes the study should demonstrate that the intervention was representative of that in use in the source population.<br>The question should be answered no if, for example, the intervention was undertaken in a specialist centre unrepresentative of the hospitals most of the source population would attend.	1	1	1	1	1	1	1	1

	**Internal validity - bias**								
14	*Was an attempt made to blind study subjects to the intervention they havereceived?* For studies where the patients would have no way of knowing whichintervention they received, this should be answered yes.	1	1	1	1	1	1	1	1
15	*Was an attempt made to blind those measuring the main outcomes of theintervention?*	1	1	1	1	1	1	1	1
16	*If any of the results of the study were based on "data dredging", was this made clear?* Any analyses that had not been planned at the outset of the study shouldbe clearly indicated. If no retrospective unplanned subgroup analyses were reported, then answer yes.	1	1	1	1	1	1	1	1
17	*In trials and cohort studies, do the analyses adjust for different lengths offollow-up of patients, or in case-control studies, is the time period between the intervention and outcome the same for cases and controls?* Where follow-up was the same for all study patients the answer should yes. If different lengths of follow-up were adjusted for by, for example, survival analysis the answer should be yes. Studies where differences in follow-up are ignored should be answered no.	1	1	1	1	1	1	1	1
18	*Were the statistical tests used to assess the main outcomes appropriate?* The statistical techniques used must be appropriate to the data. For example non- parametric methods should be used for small sample sizes. Where little statistical analysis has been undertaken but where there is no evidence of bias, the question should be answered yes. If the distribution of the data (normal or not) is not described it must be assumed that the estimates used were appropriate and the question should be answered yes.	1	1	1	1	1	1	1	1
19	*Was compliance with the interventions reliable?* Where there was non compliance with the allocated treatment or where there was contamination of one group, the question should be answered no. For studies where the effect of any misclassification was likely to bias any association to the null, the question should be answered yes.	1	1	UTD	1	1	1	1	1
20	*Were the main outcome measures used accurate (valid and reliable)?* For studies where the outcome measures are clearly described, the question should be answered yes. For studies which refer to other work orthat demonstrates the outcome measures are accurate, the question should be answered as yes.	1	1	1	1	1	1	1	1

	**Internal validity - confounding (selection bias)**								
21	*Were the patients in different intervention groups (trials and cohort studies) or were the cases and controls (case-control studies) recruitedfrom the same population?*<br>For example, patients for all comparison groups should be selected fromthe same hospital. The question should be answered unable to determinefor cohort and case- control studies where there is no information concerning the source of patients included in the study.	1	1	0	1	0	1	1	0
22	*Were study subjects in different intervention groups (trials and cohort studies) or were the cases and controls (case-control studies) recruitedover the same period of time?*<br>For a study which does not specify the time period over which patients were recruited, the question should be answered as unable to determine.	1	1	1	1	1	1	1	0
23	*Were study subjects randomised to intervention groups?*<br>Studies which state that subjects were randomised should be answered yes except where method of randomisation would not ensure random allocation. For example alternate allocation would score no because it ispredictable.	1	0	1	1	1	1	1	0
24	*Was the randomised intervention assignment concealed from bothpatients and health care staff until recruitment was complete and irrevocable?*<br>All non-randomised studies should be answered no. If assignment wasconcealed from patients but not from staff, it should be answered no.	1	0	1	1	1	1	1	0
25	*Was there adequate adjustment for confounding in the analyses fromwhich the main findings were drawn?*<br>This question should be answered no for trials if: the main conclusions ofthe study were based on analyses of treatment rather than intention to treat; the distribution of known confounders in the different treatment groups was not described; or the distribution of known confounders differed between the treatment groups but was not taken into account in the analyses. In non- randomised studies if the effect of the main confounders was not investigated or con- founding was demonstrated butno adjustment was made in the final analyses the question should be answered as no.	1	1	1	1	1	1	1	1
26	*Were losses of patients to follow-up taken into account?*<br>If the numbers of patients lost to follow-up are not reported, the questionshould be answered as unable to determine. If the proportion lost to follow-up was too small to affect the main findings, the question should be answered yes.	1	UTD	1	1	1	1	1	1

	**Power**								
27	*Did the study have sufficient power to detect a clinically important effect where the propability value for a differerance being dure to chance is less than 5%?*<br>Sample size was calculated to detect a difference of x% and y%.	1	0	0	0	1	0	0	0
**Total Score**		**26/28**	**24/28**	**25/28**	**27/28**	**27/28**	**27/28**	**27/28**	**21/28**
